# Factors associated with homecare coordination and quality of care: a research protocol for a national multi-center cross-sectional study

**DOI:** 10.1186/s12913-021-06294-7

**Published:** 2021-04-06

**Authors:** Nathalie Möckli, Michael Simon, Carla Meyer-Massetti, Sandrine Pihet, Roland Fischer, Matthias Wächter, Christine Serdaly, Franziska Zúñiga

**Affiliations:** 1 Nursing Science, Department of Public Health, University of Basel, Bernoullistrasse 28, CH-4056 Basel, Switzerland; 2 Nursing Research Unit, Inselspital Bern, Freiburgstrasse 18, CH-3010 Bern, Switzerland; 3 Department of Pharmaceutical Sciences, Clinical Pharmacy & Epidemiology, c/o University Hospital Basel, Spitalstrasse 26, CH-4031 Basel, Switzerland; 4 Institute of Primary Health Care (BIHAM), University of Bern, Mittelstrasse 43, CH-3012 Bern, Switzerland; 5 University of Applied Sciences and Arts of Western Switzerland, School of Nursing, Route des Arsenaux 16a, CH-1700 Fribourg, Switzerland; 6 Centre for Primary Health Care, University of Basel, Rheinstrasse 26, CH-4410 Liestal, Switzerland; 7 Institute for Business and Regional Economics IBR, Lucerne University, Zentralstrasse 9, CH-6002 Luzern, Switzerland; 8 serdaly&ankers snc, Route de Florissant 210, CH-1231 Conches, Switzerland

**Keywords:** Care coordination, Delivery of health care, Health services research, Home care services, Nursing administration research, Quality of care

## Abstract

**Introduction:**

The persistent fragmentation of home healthcare reflects inadequate coordination between care providers. Still, while factors at the system (e.g., regulations) and organisational (e.g., work environment) levels crucially influence homecare organisation, coordination and ultimately quality, knowledge of these factors and their relationships in homecare settings remains limited.

**Objectives:**

This study has three aims: [1] to explore how system-level regulations lead to disparities between homecare agencies’ structures, processes and work environments; [2] to explore how system- and organisation-level factors affect agency-level homecare coordination; and [3] to explore how agency-level care coordination is related to patient-level quality of care.

**Design and methods:**

This study focuses on a national multi-center cross-sectional survey in Swiss homecare settings. It will target 100 homecare agencies, their employees and clients for recruitment, with data collection period planned from January to June 2021. We will assess regulations and financing mechanisms (via public records), agency characteristics (via agency questionnaire data) and homecare employees’ working environments and coordination activities, as well as staff- and patient-level perceptions of coordination and quality of care (via questionnaires for homecare employees, clients and informal caregivers). All collected data will be subjected to descriptive and multi-level analyses.

**Discussion:**

The first results are expected by December 2021. Knowledge of factors linked to quality of care is essential to plan and implement quality improvement strategies. This study will help to identify modifiable factors at multiple health system levels that might serve as access points to improve coordination and quality of care.

**Supplementary Information:**

The online version contains supplementary material available at 10.1186/s12913-021-06294-7.

## Introduction

**Fig. 1:**
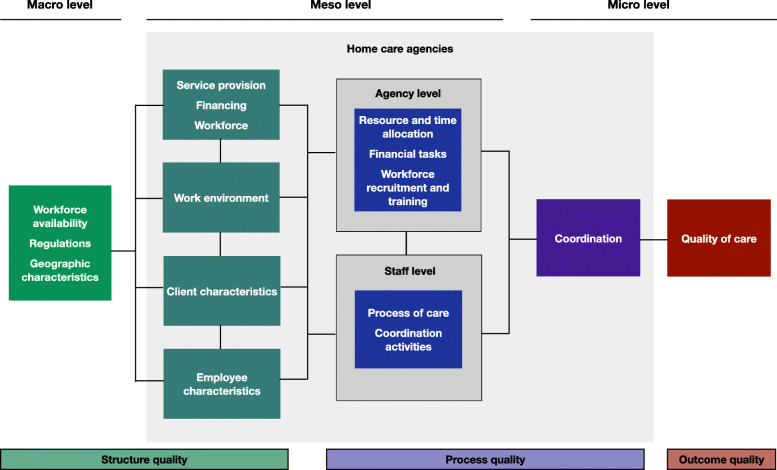
Conceptual framework

In 2018, for the first time in history, persons aged 65 years or older outnumbered children under five globally. Demographic aging will continue for some time: by 2050, in Northern North America and Europe, one person in four is expected to be 65 years or older [1]. By that time, current estimates indicate that the global population of older old persons (≥ 80 years) will have climbed from its 2019 level of 143 million to 426 million— nearly 300% the current fig [1].

As age rises, so do the prevalence of chronic conditions and multimorbidity (which affect more than 50% of those over 65), forcing many persons to become long-term care dependent [2–4]. Even when care-dependent, though, most prefer to live in their own homes as long as possible [4, 5]; and homecare is normally a cost-effective alternative to inpatient or residential care [6]. Therefore, care is shifting progressively from institutional to homecare settings [4, 7].

In Switzerland, homecare encompasses services delivered in the patient’s own home for the purpose of promoting, maintaining, or restoring health or minimizing the effects of illness and disability [8]. In 2017, Swiss homecare agencies provided services to over 350′000 clients, almost all (99%) of whom received long-term care; 70% were over 65 years of age [9]. As the population of people in that age range is growing, homecare has recently become the fastest-growing segment of Switzerland’s healthcare sector [6, 9]. Over the decade starting in 2021, keeping pace with projected care requirements will require a 57% increase in trained care providers [10].

Although health systems are being adapted to strengthen primary care and meet the complex long-term care needs of clients, the current focus on acute care hampers providers’ ability to keep pace with these increases in demand [6, 11]. The main reason for this shortfall is the fragmentation of healthcare delivery, with inadequate information flow leading to inefficient coordination and collaboration [11, 12]. This lack of care coordination also poses a major challenge to the quality of homecare services, as it can lead to negative client outcomes (e.g., health deterioration), unnecessary or incorrect treatment and wasted resources (e.g., duplication of diagnostic tests) [6, 7, 13–15]. McDonald, Sundaram et al. [16] define care coordination as "the deliberate organization of patient care activities between two or more participants (including the patient) involved in a patient’s care to facilitate the appropriate delivery of health care services. Organizing care involves the marshaling of personnel and other resources needed to carry out all required patient care activities, and is often managed by the exchange of information among participants responsible for different aspects of care" (p.41).

Viewed as a process, care coordination is most necessary to manage all transitions between care providers, thereby bridging any gaps between the client and the health care system. These might involve changes between individual professionals, teams or settings, or any other points when changes in client care are necessary [17, 18].

### Care coordination in homecare

Although homecare is interdependent with other care services, and homecare workers typically collaborate with various care providers (e.g., informal caregivers, general practitioners, social workers) [13, 19], homecare coordination is often provided on an unstructured and voluntary basis by homecare workers [13, 20]. In addition, care coordination in homecare is more challenging than in institutional settings (e.g., hospitals) [13, 21]. Homecare is non-continuous (e.g., with daily or weekly visits) and often augments the efforts of informal caregivers. Combined with relatively rare physician contact and a rather high administrative burden per hour of contact—especially for reimbursement—these characteristics limit homecare workers’ ability to ensure necessary care [21–23].

Lack of care coordination in homecare also hampers healthcare delivery in other ways. Baker, Flintoft et al. [24] found that, in homecare, medication-related adverse events were mostly related to inconsistent care coordination. Clients also attributed issues such as conflicting care plans or medication mismanagement to a general lack of reliable care coordination [25]. And 33% of healthcare patients experience primary care coordination gaps, including conflicting information, lack of availability of tests or records, or uninformed healthcare providers [26].

On the other hand, compared to homecare clients receiving usual long-term care, those receiving specifically coordinated care report reduced pain, better cognitive functionality and increased participation in activities of daily living [27]. And in Spain, recent healthcare reforms both subsidized homecare and introduced care coordination programs, which significantly reduced homecare clients hospital admissions [28].

### Factors associated with coordination in homecare

When elaborating factors associated with care coordination, the entire health system must be taken into consideration [13, 29, 30]. In developing our conceptual framework, as recommended by the WHO, one of our first steps was to divide the health care system into three distinct levels (the macro, meso, and micro levels) [29]. The macro level is where government and policy decisions are made; the meso level includes organizations such as homecare agencies; and the micro level is where client-care worker interactions occur [29]. Failure of care coordination can occur on each of these levels. The implementation of accurate strategies to enhance coordination first requires the identification of factors associated with coordination across all three system levels, as these are all interdependent [31].

As a second step, we incorporated Donabedian’s model of quality, which specifies three categories of quality: *structure quality*, *process quality* and *outcome quality*. *Structure* deals with the characteristics of the care provision setting, *process* includes all relevant tasks performed by professionals or clients, and *outcome* refers to those tasks’ effects or impacts on clients [32]. Finally, in order to establish the framework’s content (cf. Figure 1), we searched the literature for factors associated with care coordination and/or outcome quality, including homecare expert opinions. The following sections present the results of that search.

#### Macro level – structure quality

At the macro level, we identified three structural aspects with impacts on coordination and quality of care: *workforce availability*, *regulations* and *geographic characteristics*.

Regarding ***workforce availability***, together with a general nursing shortage, a constant increase in demand for staff and a lack of interest among younger nurses regarding homecare can lead not only to a severe lack of qualified staff, but also to a range of corresponding issues, especially regarding quality of care [33–36].

Governance varies widely between and within countries [13]. Governments steer homecare by setting ***regulations*** such as quality standards, client co-payments and eligibility criteria for homecare service use [13]. Poorly designed national (macro-level) legislation can unintentionally damage homecare workers’ work environments, leading indirectly to cuts in quality of care and its coordination, or directly to care coordination deterioration [37, 38]. Macro-level policies also affect the meso level with respect to working hours, full versus part-time work, and employment conditions and opportunities [39].

As a macro-level tool to influence structural quality, regulation affects both structure and process quality at the meso and micro levels. One example of unsuccessful macro-level policy occurred in Canada, where healthcare restructuring has led to heavier homecare workloads and increasingly complex cases (i.e., unstable clients with unpredictable outcomes), causing many nurses to feel overworked and generally stressed [40]. Rudoler, Peckham et al. [38] highlighted a number of these primary care reforms’ unintended effects (e.g., ineffective incentives, failure to connect sectors/organizations) that hamper progress towards coordinated care. Additionally, Norman, Wade et al. [37] found that patients’ out-of-pocket costs and eligibility criteria were major barriers to coordination.

Regarding ***geographic characteristics,*** two systematic reviews found more problems in rural than in urban regions concerning meso-level factors such as trouble filling job vacancies, overloading of local professionals, longer travel times between clients and insufficient availability of resources, e.g., inadequate equipment and facilities. Consequences included reductions in the quality of care (particularly individuals not receiving the care they needed) [41, 42]. However, city dwellers did not necessarily fare better. Smith, Anderson et al. [43] found that, compared to homecare agencies in rural locations, those in urban locations in the U.S. actually tended to score lower regarding clinical outcome measures and client experience.

From a macro-level perspective, failures of care coordination become apparent when fragmentation of health services (e.g., missing, redundant or simply wrong service provision) results in clients suffering adverse clinical incidents [17]. However, to date little information is available on how the macro-level factors influence either the meso-level operation of homecare agencies or the micro-level coordination of their services with those of other care providers.

#### Meso level – structure quality

In our model, meso-level structure quality applies to service provision, financing and workforce, the work environment and the characteristics of homecare agencies’ clients and employees.

Considering ***service provision,*** Dalby and Hirdes [44] found that homecare agencies serving smaller populations achieved higher overall quality of care. Also, clients who received their first homecare visits during weekends were more likely to suffer adverse events, e.g., injuries from falls, wound infections and medication errors. However, regular weekend visits by homecare workers were associated with a decrease in such events [45].

As for ***financing***, how homecare agencies are financed appears to play an important role in relation to care coordination, as coordination requires time and personnel [46]. Studies in the U.S. indicate that financing models had an impact, with for-profit agencies scoring lower on overall quality measures [47, 48] and showing higher risks of client rehospitalization [47, 49] than non-profit agencies. In Canada, fixed multi-year service agreements resulted in understaffing and increased workload [40].

Regarding the ***workforce***, Smith, Anderson et al. [43] found that agencies with higher numbers of homecare aides per 100 visits scored lower on clinical outcome measures and client experience. Furthermore, higher proportions of licensed practical nurses and nurse aides, as opposed to registered nurses, were associated with lower care quality and higher hospitalization rates [48].

As for the ***work environment****,* one study found that, in homecare workers’ view, a reduced workload, frequent team meetings and increased management and supervision time were crucial elements for good care coordination [50]. Similarly, Swedish study in homecare assistant nurses found that work environment characteristics such as transformational leadership, peer support and job control correlated with higher quality of care [51]; a U.S. study among homecare nurses found associations between better organizational support and higher overall care quality, fewer medication errors and less uncontrolled pain [52]; and a scoping review identified several meso-level factors, such as peer support, role clarity, manageable workload and collaboration that influence optimal homecare nursing [40].

Other studies have shown that ***client characteristics*** such as age, co-morbidities, gender (inconclusive in which direction), depression, cognitive and functional impairment, low client compliance and living alone increase the risk for adverse events at home [15, 53] and were associated with higher rehospitalization rates [54].

Studies on ***employee characteristics*** are scarce. However, one found nonsignificant relationships between homecare employee characteristics such as age and job tenure with adverse events [45].

We were unable to identify any relevant studies focusing on the various meso-level elements of structural quality in relation to micro-level care coordination.

#### Meso level – process quality

In constructing our conceptual framework, for meso-level processes we differentiated those at the agency level from those at the staff level. The agency level includes resource and time allocation, financial tasks, and workforce recruitment and training; the staff level includes care and coordination activities.

One US study named adequate ***resource and time allocation*** factors such as opportunities to interact and communicate intra- and inter-professionally, as instrumental to the improvement of homecare nursing [40], including reduction of hospital readmission rates [55]. Nevertheless, a qualitative US study found that homecare nurses often had difficulty accessing medical information, leading to the use of more time than allocated [56]. The same study reported that homecare nurses commonly had to make care decisions based on the observations of nursing assistants, who have less education and training, while more and more tasks are assigned to them [56]. In addition, agencies assigning smaller numbers of cases to each case manager performed better regarding overall quality of care [44]. While supporting evidence is currently scarce, this strongly suggests that time and other resources for effective information exchange and care planning are important factors for care coordination [46].

Regarding homecare agencies’ ***financial tasks***, enabling and incentivizing them to cover care coordination expenses is fundamental. Where problems with payment occur, they have the opposite effect [27, 46]. To date, we have not found any studies exploring how care quality or care coordination is affected by homecare agencies’ financial tasks, e.g., seeking reimbursement, determining or negotiating the amount of time billable to health insurers, or the planning or realization of cost saving measures.

Concerning ***workforce recruitment and training***, a qualitative study reported that a trained and available workforce is essential for sustainable care coordination; therefore hiring and retaining workers are also vital concerns [46]. Furthermore, qualitative studies have found that knowledge of the system and the necessary roles and responsibilities is an important element of effective care coordination [18, 57]. According to the scoping review of Masotti, McColl et al. [15], low team experience, training and knowledge, as well as inadequate patient monitoring/assessments, were frequently reported as factors contributing to adverse events in homecare. As a result, training opportunities were seen as crucial for care coordination by homecare workers [50]. However, to our knowledge, no studies have yet explored these various elements’ associations with care coordination in the homecare setting.

A deeper understanding of the ***process of care*** is crucial to determine necessary care coordination activities. These include assessing needs, defining goals, proactively planning care, and monitoring and responding to change [17, 57]. To effectively coordinate care, a qualitative study found that care workers need both to understand their clients (e.g., details of their conditions, needs and preferences) and to empower them (e.g., how to use health services, manage their health) [18]. Each of these reflects a step in the process of care.

In our model, ***coordination activities*** can be understood as those undertaken by participating care providers in managing dependencies [58]. Identified activities include establishing accountability or negotiating responsibilities, communicating and facilitating transitions with the various care providers, linking community resources and aligning resources with client needs [17]. A recent US homecare study found that the most common coordination activities are follow-up with clients, assistance in completing applications and provision of service referrals [37]. Another is communication. A scoping review found communication issues the most commonly reported factors related to adverse events [15]. More specifically, the absence of standardized communication between team members has been strongly associated with medication-related events [24].

From a meso-level point of view, care coordination gaps become apparent when clients are directed to inappropriate health services or experience negative health outcomes due to inadequate handover or information exchange [17].

#### Micro level – process quality

In our model, care ***coordination*** denotes “effective management of dependencies between subtasks, resources (e.g. equipment, tools, etc.) and people” [58]. To achieve overall care goals, care coordination focusses on facilitating high quality care provision across multiple providers to meet the client’s needs and preferences [17]. Therefore, our framework presents coordination as a micro-level driver of process quality. On this level, care coordination failures often highlight additional efforts clients or informal caregivers have to make to ensure information flow or to meet care needs during transitions, i.e., shifts in responsibility [17]. If both macro- and meso-level factors facilitate (micro-level) care coordination, improvements can be expected not only in coordination but in care outcomes.

#### Micro level – outcome quality

Campbell, Roland et al. [59] define ***quality of care*** as a measure of individuals’ ability to “access the health structures and processes of care which they need and … [the extent to which] the care received is effective” (p.1614). With successful care coordination, higher quality of care can be achieved, e.g., in terms of reduced hospitalizations, improved clinical outcomes and higher levels of client satisfaction [60]. A study in the primary care setting showed that enhanced care coordination reduces the likelihood of hospitalizations or emergency room visits [26]. However, the specific association between care coordination and quality of care in the homecare setting remains unclear.

Considering the interplay between the micro, meso and macro health system levels, a system-wide overview is useful in evaluating or planning strategies to enhance coordination and improve quality of care. Detailed knowledge of how a system is performing makes it possible to select targets both for quality improvement and for investment [14]. Therefore, it is essential to explore how the three system levels interact. Although a number of qualitative studies have explored coordination-related factors, to our knowledge, very few quantitative studies have assessed macro- and meso-level factors’ associations with care coordination. To develop and implement successful strategies to improve care coordination, knowledge of these relationships on every level is essential.

## Methods

### Aim

As little is known about the dynamic interplay between macro-, meso- and micro-level factors regarding care coordination and, in the end, quality of care in homecare, the following overall aims will be pursued:

1. to explore how macro-level factors are associated with (meso level) homecare agency structures and processes;
2. to explore macro- and meso-level factors’ associations with (micro level) care coordination; and
3. to explore care coordination’s associations with (micro level) quality of care.

### Study design and setting

The proposed study is a national multi-center cross-sectional survey in the Swiss homecare setting.

Of Switzerland’s 1020 homecare agencies, 577 are non-profit and 443 for-profit agencies [9]. Non-profit agencies care for roughly 80% of all homecare clients. They are larger on average than their for-profit counterparts, with an average of 31 full-time-equivalent staff (FTEs), versus 9 for for-profit agencies [9]. Many homecare employees work part-time, with a mean employment rate of 45% in 2017 [9].

Homecare in Switzerland is funded by three sources: 1) the mandatory health insurance system; 2) client copayments; and 3) public funding of residual costs. Depending on the nursing tasks performed, insurers pay an hourly amount specified by the federal government [61]. The 26 Cantons of the Swiss Confederation, which have a relatively high degree of autonomy regarding healthcare decisions, are responsible for regulating client copayments and public funding. In some cantons, no copayments are required; in others clients pay up to 20% of the health insurance expenditures and up to a maximum of 15.95 CHF (approximately 15 Euro) per day of homecare services as defined by the federal government [61]. Requirements for and the extent of public funding also differ considerably between cantons [62].

### Sample

Our sample will consist of homecare agencies, including their homecare workers, their clients, and the clients’ informal caregivers. For this purpose, a three-stage sampling procedure will be carried out.

First, we will use a stratified random sample of ***homecare agencies***. Agencies will be pooled in the seven geographic regions used by the Swiss Federal Statistical Office [63] and stratified for each of those regions according to their profit status (non-profit/ for-profit). Only agencies with ten or more salaried employees will be included. Self-employed homecare nurses will be excluded. A formal power analysis is difficult in this context as many parameters, e.g., cluster effects of coordination outcomes, are unknown. For a multilevel analysis where the interest is mostly focused on fixed parameters, at least 30 groups of at least 30 individuals will be necessary for reliable results [64]. If there are strong interests in cross-level interaction, the number of groups should be larger—roughly 50 groups of 20 individuals per group. Our interest will be in cross-level interactions (aims 1 and 2) and fixed parameters (aim 3). The target sample size will be 107 homecare agencies, with 15% of the total sample size in each geographic region being non-profit and 15% for-profit agencies. Regarding homecare agency sizes in Switzerland, 50% of non-profit and 75% of for-profit ones represent fewer than ten FTE positions. Considering an average employment rate of 45%, excluding agencies with fewer than 30 employees would leave fewer than 50%. To overcome this problem, despite our knowledge that reducing the minimum number of FTEs would weaken the study’s statistical power, we have chosen to include agencies with a minimum of ten employees.

Second, all ***homecare workers*** within each of the participating agencies who fulfill the following criteria will be invited to participate: 1) aged 18 years or older; 2) employed by the participating agency for at least 3 months; and 3) able to understand written German, French or Italian. With a mean of 44 homecare workers per agency and a response rate of 60%, we expect to achieve a sample size of approximately 3060 participants.

Third, within each of the participating agencies, 50 ***homecare clients*** (and their informal caregivers) will be randomly selected and invited to participate in our questionnaire survey. For agencies with fewer than 50 clients, all clients will be invited. Only clients aged ≥60 years and receiving nursing care will be included. We anticipate that roughly 30% of participating agencies will have fewer than 50 clients. Assuming a mean of 32 homecare clients per agency, a response rate of 30% would result in a final sample size of 1113 participants. For each participating client, a relative who accompanies him or her in everyday life is also invited to fill out the questionnaire for informal caregivers. If half of all invited clients pass on the questionnaire to their informal caregiver, with a 30% response rate, we expect a final sample size of roughly 550 participants.

### Instruments and measurements

**Fig. 2:**
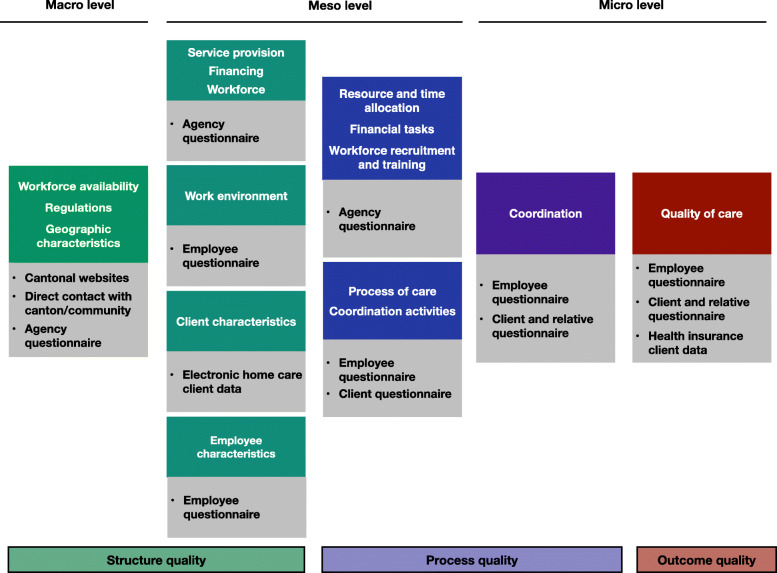
Measurements used for the three system levels

To answer our research questions, data will be gathered from various sources. Figure 2 gives an overview of the measurements planned for the different levels. Questionnaires were iteratively developed in close collaboration with stakeholders (e.g., homecare nurse experts, managers, clients and their informal caregivers, homecare associations and political representatives). As a first step, an overview of existing scales measuring the different elements of interest was created. As well as focus group interviews with homecare workers, clients and informal caregivers, various group discussions and individual interviews were conducted with diverse stakeholders to discuss the questionnaires’ key content and possible scales. Our decisions of which items to include and which scales to use were based on the research group’s discussions of the interviews’ results. The four questionnaires were developed first in German, then translated into French and Italian. Validated translations were used when possible. The entire questionnaires were then back-translated into German and checked for inconsistencies, which were then discussed with bilingual local homecare workers (i.e., managers, nurses), clients and informal caregivers, then linguistically adjusted if necessary. After translation, using cognitive interviews, the questionnaires were pretested in each of Switzerland’s three language regions.

For an overview of the variables measured at each level, see Table 1 (below).

#### Macro level – structure quality

For structure quality on the macro level, three separate data sources will be used: 1) the websites of the cantons; 2) direct contact with cantons/municipalities; and 3) an agency questionnaire.

For each participating agency, data on public funding and reimbursement regulations will be collected, including those concerning client copayments [61] and residual financing, which must be provided either by cantons, by municipalities or by both. We will also collect data on the apportionment of residual financing, requirements for reimbursement (e.g., operating licenses, service agreements, cost calculation standards, required assessment tools) and methods of financing (e.g., shortfall warranty, paid hours of performance, standard vs. total costs). Geographic characteristics such as population size, numbers of physicians, pharmacies and hospitals will be recorded as appropriate. To assess workforce availability, we will ask agency managers about their perceptions regarding challenges to recruitment of qualified nursing personnel.

#### Meso level – structure quality

For meso-level structure quality, three data sources will be used: 1) an agency questionnaire; 2) an employee questionnaire; and 3) electronic homecare client data.

For service provision, we will include agency size (number of FTEs, total hours of care provided in 2020), range of services and availability of services. Financing will be classified according to profit/non-profit status, percentage of financial contributions from all contributors and service agreements with cantons or municipalities. Regarding the workforce*,* we will assess the number of salaried employees at the time of data collection and the staff turnover rate. We will also measure staffing and skill mix, which are evaluated according to the percentage of the total number of care workers who are registered nurses, and the number of registered nurse visits per 50 home visits.

The work environment will be measured with validated instruments (e.g., the Safety Attitude Questionnaire [65, 66], Copenhagen Psychosocial Questionnaire [67]), and several self-developed scales and items. Table 1 (below) provides an overview of the variables; Appendix A provides detailed information on the employee questionnaire measurements and scales (see Additional file 1).

Client characteristics will be assessed using data extracted from the homecare agency database ADUA (*Administrative Daten und Anfrage* (translation: “administrative data and query”)): year of birth (to calculate age), gender, living situation, place of care, minutes of professional care per visit, service intervals, types of services and whether services are covered by health insurance. Additional information, such as prior hospitalizations and the client’s care needs (e.g., regular / palliative / psychiatric) will be assessed to deduce client profiles (% of clients receiving regular care, etc.).

Employee characteristics, including age, gender, employment rate and experience, will also be assessed.

#### Meso level – process quality

Meso-level process quality will be gauged via three data sources: 1) an agency questionnaire; 2) an employee questionnaire; and 3) a client questionnaire.

Resource and time allocation data include variables such as the organization of the last three working days, regular intra- and/or interprofessional case discussions and/or team meetings, communication technologies currently in place, use of a planning system based on a reference person, and number of cases per nurse. For financing tasks, we will include criteria for reimbursement, settlement of conflicts with health insurance companies and municipalities regarding the financing of services, experienced cost pressure, the amount of time and costs not billable to health insurance, and planning and/or realization of cost-saving measures. Regarding workforce recruitment and training, we will assess the presence of nurses with case responsibilities / case managers / care managers (persons responsible and contact persons for individual clients regarding the care process or problems), as well as any provision by agencies of care worker training. We will also assess the presence of standards, checklists and guidelines for selected procedures and the availability of clear task/role descriptions.

On the staff level, evaluating the process of care includes questionnaire items asking whether interprofessional care goals and treatment plans are set, evaluated and adapted involving clients. Regarding coordination activities, from the employee perspective we will measure communication [68], accountability, predictability, common perspectives [69] and familiarity with the healthcare system. From the client perspective, we will assess communication between providers and clients [70] as well as coordination of homecare agencies [71] and the extent to which homecare nurses take up coordinator roles [72]. For detailed information regarding the measures in the client questionnaire, see Appendix B (see Additional file 1).

#### Micro level – process quality

To measure process quality on the micro level, three different data sources will be necessary: 1) an employee questionnaire; 2) a client questionnaire; and 3) a questionnaire for informal caregivers.

To measure coordination from the employee perspective, we will assess the alignment of work within the care team, the alignment of client care with nominated providers (e.g., hospitals, general practitioners) and selected types of care coordination gap. Since we have been unable to locate any scales to measure care coordination as per our definition, all necessary scales have been developed by the authors; for details see Appendix A [see Additional file 1]. From the clients’ and informal caregivers’ perspectives, we will assess the perceived overall care coordination [71] and role clarity as well as care coordination between settings [72]. Detailed information about the measures in the informal caregiver questionnaire can be found in Appendix C (see Additional file 1).

#### Micro level – outcome quality

As suggested by Hanefeld, Powell-Jackson et al. [73], we will employ three separate approaches to our development of a comprehensive understanding of the quality of care delivered, i.e., not only clinical indicators but also client and care provider perceptions must be assessed and compared. Regarding provider perceptions, studies have indicated very strong correlations between nurse-sensitive quality measures (e.g., falls, pain) and nurse-reported quality in hospitals (overall rating of the quality of patient care) [74, 75]. Therefore, they will be included in the first of our micro-level outcome quality measures, i.e., our employee questionnaire. In all, four data sources will be used: 1) an employee questionnaire; 2) a client questionnaire; 3) a questionnaire for informal caregivers and 4) health insurer billing data.

One approach to measuring outcome quality is via employees’ perceptions of quality of care, i.e., by asking them to rate their perception of the overall quality of client care (e.g., “On a scale of 1 to 10, with 1 representing “very low quality” and 10 representing “very high quality,” how do you rate the quality of client care in your own homecare agency?” [75]). A second approach is to assess the quality of care perceived by clients and their informal caregivers, i.e., asking them to rate the overall quality of homecare they have received [70] as well as other health care service utilization by clients, such as their number of hospitalizations, emergency room visits and doctor visits (general practitioners and specialists) [76]. Our third approach is to obtain anonymized billing data from a sample of health insurance companies. These allow accurate calculation of the number of unplanned hospitalizations, visits to the emergency department and visits to the general practitioner over the last 12 months.

Table 1**.** Overview of the variables measured at each level.

**Table 1:** Overview of the variables measured at each level

Topic	Level	Domain	Variable
Structure quality	Macro	Workforce availability	Recruitment situation for nursing and care staff
		Regulations	Reimbursement regulations (health insurance, client co-payments, residual financing and methods of financing)
			Requirements for and content of an operating license
			Requirements for and content of a service agreement
			Requirements for reimbursement
		Geographic characteristic	Catchment area (rural, suburban, urban)
			Agency’s service area (population size, numbers of physicians, pharmacies and hospitals)
	Meso	Service provision	Number of full-time equivalent posts
			Total number of clients and hours of care provided in 2020
			Range of service (e.g., nursing care, domestic tasks, meal service, specialized care)
			Availability of services (e.g., only by day, day and night, on the weekend)
		Financing	Profit status (non-profit, for-profit)
			Percentage of financial contributions from different contributors (e.g., health insurance, client, canton/municipalities)
			Obligation to supply or service agreement with municipalities and cantons
		Workforce	Numbers of full-time equivalent positions differentiated according to educational background
			Turnover rate
			Staffing and skill mix (percentage of RNs and number of visits conducted by RNs within the last 50 visits)
		Work environment	Leadership
			Perceived staffing
			Teamwork
			Workload
			Overtime
			Predictability
			Role clarity
			Role conflicts
			Social support
			Sense of community
		Client characteristics	Age
			Gender
			Living situation (e.g., alone / with partner / with children)
			Type of services used (nursing care, domestic services or both)
			Service intervals (daily / weekly / monthly)
			Services covered by health insurance
			Place of care (e.g., apartment, house)
			Minutes of professional care per visit
			Prior hospitalizations
			Care needs (e.g., regular / palliative / psychiatric)
		Employee characteristics	Age
			Gender
			Employment percentage
			Experience in their profession
			Experience in their current homecare agency
			Job / position
			Country of education
			Educational background
Process quality	Meso	Resources and time allocation	Organization of the last seven working days (e.g., number of nurses, number of visits, travel times, amount and type of services, time for coordinative and administrative work)
			Intra- and/ or interprofessional case discussions and/or team meetings
			Communication channels/technologies in place
			Planning according to a reference person system Number of cases for which each nurse is responsible
		Financial tasks	Requirements for reimbursement
			Conflicts with health insurance companies and municipalities pertaining to the financing of services
			Experienced cost pressure
			Time and costs not billable to health insurance
			Planning or realization of cost saving measures.
		Workforce recruitment and training	Presence of nurses with case responsibilities / case managers / care managers
			Provision of care worker training (e.g., regarding service availability, interprofessional care coordination)
			Presence of standards, checklists and guidelines for selected procedures (e.g., medication management, wound therapy, emergency situations)
			Clear task/role descriptions
		Process of care	Presence of interprofessional care goals
			Evaluation and adaption of care and treatment plans
		Coordination activities	Communication and information exchange
			Communication channels used
			Accountability, predictability, common perspective
			Familiarity with the healthcare system
			Communication between providers and clients (client perspective)
			Extent of coordinator role of homecare nurses (client perspective)
			Coordination through homecare agency (client perspective)
	Micro	Coordination	Alignment of work within the care team
			Alignment of client care with nominated providers
			Care coordination gaps (from employee and client perspective)
			Overall rating of coordination (from client and relative perspective)
			Role clarity and coordination between settings (from client perspective)
Outcome quality	Micro	Quality of care	Rating of care provided by the agency (from employee, client and relative perspective)
			Health care service use

*RN* registered nurse

### Data collection

Data collection will take place from January 2021 until June 2021. Before data collection begins, each agency will choose a contact person who will be responsible for internal distribution of the questionnaires to the employees, clients and informal caregivers. At least 2 months in advance, that person will be informed in detail about the data collection procedure. Each agency will be given 9 weeks to fill out the questionnaires.

The agency questionnaire will be delivered as an interactive pdf document and filled out by the management. Employees will receive paper-based questionnaires, each containing a return envelope addressed directly to the Institute of Nursing Science (INS). By preventing the collection of questionnaires by agencies, this will ensure confidential treatment of data. In line with data protection requirements, paper-based questionnaires will be distributed by homecare agencies to selected clients and their informal caregivers. The research team will support one person from the administration of each agency in randomly selecting clients without requiring access to client information. Every envelope will contain two questionnaires, one for the client and one for their relative/informal caregiver, and two prepaid return envelopes addressed to the Institute of Nursing Science (INS). Again, this is to avoid the collection of questionnaires by agencies. The clients are asked to give the relative questionnaire to the person who supports them in their daily life. To minimize response bias, homecare workers will be informed that they are not allowed to fill out the questionnaires with clients. Support by relatives is possible. We will send a request to each agency contact person for the participating homecare clients’ relevant ADUA data. These will have to be exported and transmitted to the INS in anonymized and aggregated format. We will also request the relevant billing-related information from each participating insurer. Again, we will instruct them fully regarding the appropriate data handling procedures, including the use of an encrypted data transmission platform.

### Patient and public involvement

To enhance the quality of this research, we will follow the INVOLVE standards as guidelines to work with public and patient involvement [77–79]. A stakeholder group, including representatives of various fields, e.g., research, practice, politics and professional associations, as well as a client, has been established to provide input and support throughout the study. In addition, clients, informal caregivers and homecare workers will be invited to discuss various aspects of the research process (e.g., questionnaire development and layout, design of information material, reporting and visualization of the result).

### Data analysis

Statistical analyses will be conducted using the R version 3.X statistics programming environment [80]. First, data will be assessed for plausibility. Descriptive statistics will then be computed to summarize frequencies and percentages or means/medians with standard deviations/IQRs as appropriate. Data will be checked for missing values, floor and ceiling effects, normal distribution, and outliers. Items with more than 90% agreement or more than 5% missing answers will be checked for subgroup differences (e.g., professional background, professional experience, age). To assess the internal structure or inter-item consistency (e.g., Cronbach’s α), psychometric analyses will be performed on all scales used. Depending on the data quality, appropriate strategies for handling missing data (e.g., multiple imputation) will be incorporated.

To explore relationships between the different levels, we will begin by assembling clusters of homecare agencies with similar policies / funding mechanisms. In a second step, we will use multiple regression analyses to investigate the associations between macro-level regulatory factors and meso-level homecare agency structures or processes (aim 1). To examine which regulatory factors on the macro and organizational factors on the meso level are linked with micro-level coordination (aim 2), and which connect coordination to quality of care (aim 3), we will use multilevel analyses.

After completion of this research project, the data will be stored for 10 years in CSV format in the Information Technology Services (ITS) department of the University of Basel. For metadata, including the description of the document, the study, the variables and the files, the Data Documentation Initiative (DDI) standard, an international standard for describing observational data, will be applied [81]. Metadata will be stored in an xml file. Due to the sensitive and confidential nature of the agency, employee, client and relative data, non-disclosure agreements will be signed. None of our collected data will be openly accessible; however, with the consent and assistance of the principal investigator, re-use of the anonymous materials will be possible.

## Discussion

As the proposed study will be the first national survey to explore macro-, meso- and micro-level factors influencing coordination and quality of care in the Swiss homecare setting, it will provide valuable insights into this increasingly important branch of healthcare. In addition to gaining the first insights at this level into homecare quality in Switzerland, we expect to identify factors related to coordination and quality in homecare on every level of the health care system. This knowledge will help to develop and implement targeted strategies to enhance coordination. This research project’s first results are expected by the end of 2021. All study results will be published in peer reviewed journals.

One notable weakness of this research project is its cross-sectional design, which does not allow inference of causal relationships. However, as this is an explorative project with a representative sample, it is possible to make generalized statements about factors related to quality of care and coordination. Additionally, our study design removes any opportunity of us to control the environment while participants complete their questionnaires, and could increase recall bias. However, it is hoped that supplying a pre-stamped envelope for client and employee questionnaires will minimize the pressure towards social desirability bias.

The results of this project will support policy makers and homecare administrators in developing coordination interventions in homecare settings across Switzerland. In addition to improving need-oriented care provision, this study’s findings regarding increased coordination of the various service providers’ activities will very likely help reduce resource waste. Equally importantly, they provide a firm foundation upon which to develop a range of interventional, implementation science and quality improvement projects in homecare.

## Supplementary Information

**Additional file 1.** Questionnaire measurements. Description of employee, client and relative questionnaire measurements.

## Data Availability

The datasets used and/or analyzed during the current study are available from the corresponding author on reasonable request.

## Abbreviations

ADUA: Administrative Daten und Anfrage (translation: “administrative data and query”)
CHF: Swiss Francs
FTE: Full-time-equivalent
RN: Registered nurse
