# Supplementary material for: Factors associated with homecare coordination and quality of care: a research protocol for a national multi-center cross-sectional study
Source: BMC Health Serv Res. 2021 Apr 6;21:306. doi: 10.1186/s12913-021-06294-7 (PMC8025374; doi:10.1186/s12913-021-06294-7)
Supplement: Supplementary file 1 — Additional file 1. Questionnaire measurements. Description of employee, client and relative questionnaire measurements. [file 12913_2021_6294_MOESM1_ESM.docx]

# Additional file 1

**Appendix A.** Employee questionnaire measurements

| **Variables** | **Instrument used**  **(Reference)** | **Number of items (Anchor of answer options)**  **Scale calculation / Cronbach’s α** | **Meaning of score** | **Example of items** |
| --- | --- | --- | --- | --- |
| **Work environment** | |  |  |  |
| Leadership | PES-NWI  (1) | 5 (1 = strongly disagree, 4 = strongly agree)  mean over all items / .88 in SPOT (2) | higher values indicating better performing leadership | “Supervisors use mistakes as learning opportunities, not criticism.” |
| Staffing | PES-NWI  (1) | 3 (1 = strongly disagree, 4 = strongly agree)  mean over all items / .74 (.70 in SPOT) | higher values indicating higher staffing adequacy | “There is enough staff to get the work done.” |
| Teamwork | SAQ  (3, 4) | 7 (1 = disagree strongly, 5 = agree strongly) with answer option "not applicable"  mean over all items / .65 (.83 in SPOT(2)) | higher values indicating better teamwork | “Input is well received in this team.” |
| Workload | NASA TLX  (5) with one additional self-developed item | 6 (0 = low, 20 = high)  mean over all items / .67 in SPOT (not published) | higher values indicating higher perceived workload | How much mental and perceptual activity was required (e.g., thinking, deciding, calculating, remembering, looking, searching)? |
| Overtime | from previous studies (SHURP, RN4Cast)  (6, 7) | 1 (1 = never, 5 = almost every shift)  N/A | higher values indicating higher amount of overtime | How often do you have to work overtime more than 30 minutes? |
| Predictability | COPSOQ III  (8) | 2 (0 = to a very small extent 4 = to a very large extent)  mean over all items / .73 | higher values indicating higher predictability | Do you receive all the information you need in order to do your work well? |
| Role clarity | COPSOQ III  (8) | 2 (0 = to a very small extent 4 = to a very large extent)  mean over all items / .82 | higher values indicating higher role clarity | Do you know exactly which areas are your responsibility? |
| Role conflicts | COPSOQ III  (8) | 2 (0 = to a very small extent 4 = to a very large extent)  mean over all items / .73 | higher values indicating higher role conflicts | Are contradictory demands placed on you at work? |
| Social support from colleagues and supervisors | COPSOQ III  (8) | 4 (0 = never, 4 = always, with answer option “don't have a superior/ colleagues”)  mean over all items / .87 resp. .81 | higher values indicating higher work-related social support | How often do you get help and support from your colleagues? |
| Sense of community | COPSOQ III  (8) | 1 (0 = never, 4 = always, with answer option “don't have a superior/ colleagues”)  NA | higher values indicating higher sense of community | Is there a good atmosphere between you and your colleagues? |
| **Staff-level processes** | |  |  |  |
| Process of care | |  |  |  |
| Care process | Self-developed and adapted from AITCS II (9) | 5 (0 = never, 4 = always)  N/A | higher values indicating more pronounced interprofessional care process | Are interprofessional client care and treatment goals defined?  Are clients and/or relatives involved in setting goals for their care? |
| Coordination activities | |  |  |  |
| Communication and Information Exchange | Adapted from CPAT (10) | 6 (1 = disagree strongly, 5 = agree strongly)  mean over all items / .84 | higher values indicating better communication and information exchange | Client concerns are addressed effectively through regular team meetings and discussion. |
| Communication channels | Self-developed | 2 (by phone, in written form with the client file, in written form via e-mail, personal, other)  N/A | communication channels frequently used | How do you communicate important information about your clients, with people, involved in the care/treatment outside your homecare team, most often? |
| Accountability, predictability, common perspective | Adapted from the integrating condition scale of Thomas, Spitzmüller et. al. (11) | 4 (1 = disagree strongly, 5 = agree strongly) | higher values indicating higher accountability, predictability, common perspective | It is clear which professionals in our care team are responsible for fulfilling certain tasks. |
| Familiarity with the healthcare system | Self-developed | 6 (1 = not at all, 5 = very well) | higher values indicating higher familiarity with the healthcare system | How well do you know the health care services in the catchment area of your homecare:  Available health or social services?  Requirements that clients must meet in order to benefit from the services? |
| **Coordination** | |  |  |  |
| Care coordination: Alignment of work | Self-developed and adapted from Zackrison (12) | 7 (1 = never, 5 = very frequently) | higher value indicating better alignment of work | Are related processes and activities for client care well harmonized with other professionals? |
| Alignment of client care with nominated providers | Self-developed | 7 (1 = never, 5 = very frequently)  N/A | higher values indicating higher satisfaction with  the alignment of client care | How often are you satisfied with the alignment of client care with the following professional groups?  E.g., physicians, hospitals |
| Care coordination gaps | Self-developed | 7 (1 = never, 5 = very frequently) / N/A | higher values indicating more frequently experienced coordination gaps | How often does it happen that...  …you receive important information about the client too late?  ...no or no current orders / prescriptions / medication lists are available? |
| Relational coordination with informal caregivers | RCS  (13) | 7 (1=Never, 5=Completely) / mean over all items / 0.86 | Higher values indicating higher relational coordination | Do informal caregivers communicate with you in a timely way about your client's support/care? |
| **Quality of care** | |  |  |  |
| Quality and safety | from previous studies and self-developed  (7, 14) | 4 (0 = very low, 4 = very high) /  N/A | higher values indicating higher quality resp. higher safety | How do you rate the quality of client care in your own homecare agency?” |

**Note.** AITCS = Assessment of Interprofessional Team Collaboration Scale, COPSOQ = Copenhagen Psychosocial Questionnaire, CPAT = Collaborative Practice Assessment Tool, HPSI = Health Professions Stress Inventory, N/A = not applicable, NASA TLX = NASA task load index, PES-NWI = Nursing Work Index’s Practice Environment Scale, RCS= Relational Coordination Scale, RN4Cast = Nurse Forecasting: Human Resources Planning in Nursing, SAQ = Safety Attitude Questionnaire, SHURP = Swiss Nursing Homes Human Resources Project, SPOT = SPitex work environment pilOT study, WLC = Work-Life Climate

**Appendix B.** Client questionnaire measurements

| **Variables** | **Instrument used**  **(Reference)** | **Number of items (Anchor of answer options)**  **Scale calculation /**  **Cronbach’s α** | **Meaning of score** | **Example of items** |
| --- | --- | --- | --- | --- |
| **Coordination activities** | | | | |
| Communication between providers and clients | HHCAHPS  (15) | 6 (1 = Never, 4 = Always or 1= Yes, 2 = No or 1 = Same day, 4 = More than 14 days, with answer option “Do not remember” or “I did not contact this agency”)  Average proportion over all items of respondents who responded “Always” and “No” and “same day”/ .70 (on request) | higher values indicating better communication between providers and clients | In the last 2 months of care, how often did home health providers from this agency keep you informed about when they would arrive at your home? |
| Coordination through homecare agency | CPCQ (16) | 4 (1=never, 5=always)  mean over all items / .80 | higher values indicating better coordination | How often did your homecare nurse seem to be communicating with your other providers? |
| Extend of coordinator role of homecare nurses | Patient perceived continuity of care from multiple clinicians (17) | 5 (1=not at all, 5=totally)  mean over all items / .87 | higher values indicating a higher presence of coordinator role | How much does your homecare nurse seem up-to-date about health care given by others? |
| **Coordination** | | | | |
| Overall rating of coordination | Adapted from CPCQ (16) | 1 (1 = Never, 5 = Always) / NA | higher value indicating better perceived coordination | In the past 2 months, how often did you feel the care you received was well coordinated? |
| Role clarity and coordination between settings | Patient perceived continuity of care from multiple clinicians (17) | 3 (1=never to 5=very often))  mean over all items / .82 | higher values indicating higher role clarity and coordination between clinics | Were there times when health professionals told you different things (that didn’t make sense together) about your health? |
| **Quality of care** |  |  |  |  |
| Rating of care provided by the agency | HHCAHPS  (15) | 1(0 = worst home health care possible, 10 = best home health care possible) / N/A | higher values indicating better care | Using any number from 0 to 10, where 0 is the worst home health care possible and 10 is the best home health care possible, what number would you use to rate your care from this homecare agency? |
| Use of health care services | Schweizerische Gesundheits-befragung 2017 and self-developed  (18) | 10 (1= Yes, 0 = No and continuous answer options) / N/A | higher values indicating higher use of services | How many times have you been to a doctor in the last 12 months? |

**Note.** CPCQ = Client Perceptions of Coordination Questionnaire, HHCAHPS = Home Health Care Survey of the Consumer Assessment of Healthcare Providers and Systems,

**Appendix C**. Informal caregiver questionnaire measurements

| **Variables** | **Instrument used**  **(Reference)** | **Number of items (Anchor of answer options)**  **Scale calculation /**  **Cronbach’s α** | **Meaning of score** | **Example of items** |
| --- | --- | --- | --- | --- |
| **Coordination** | | | | |
| Overall rating of coordination | Adapted from CPCQ (16) | 1(1 = Never, 5 = Always) / NA | higher value indicating better perceived coordination | In the past 2 months, how often did you feel the care your relative received was well coordinated? |
| Perceived effort for coordination | Self-developed | 1(0= Not at all effortful,  10= Very effortful) / N/A | higher values indicating higher effort for care coordination | Using a number from 0 to 10, please rate how effortful you feel it is to coordinate the care with all the health professionals involved? |
| Relational coordination with homecare workers | RCS  (13) | 7 (1=Never, 5=Completely) / mean over all items / 0.86 | Higher values indicating higher relational coordination | Do homecare workers communicate with you in a timely way about your relatives' support/care? |
| **Quality of care** |  |  |  |  |
| Rating of care provided by the agency | Adapted from HHCAHPS  (15) | 1(0 = worst home health care possible, 10 = best home health care possible) / N/A | higher values indicating better care | Using any number from 0 to 10, where 0 is the worst home health care possible and 10 is the best home health care possible, what number would you use to rate the care from this homecare agency? |
| Willingness to recommend homecare agency | Adapted from HHCAHPS  (15) | 1 (1= Definitely yes, 4 = Definitely no) / N/A | higher values indicating higher willingness to recommend agency | Would you recommend this homecare agency to other family members or friends if they needed home health care? |

**Note.** CPCQ = Client Perceptions of Coordination Questionnaire, HHCAHPS = Home Health Care Survey of the Consumer Assessment of Healthcare Providers and Systems, RCS= Relational Coordination Scale

# References

1. Lake ET. Development of the practice environment scale of the Nursing Work Index. Res Nurs Health. 2002;25(3):176-88. doi:10.1002/nur.10032

2. Möckli N, Denhaerynck K, De Geest S, Leppla L, Beckmann S, Hediger H, et al. The home care work environment’s relationships with work engagement and burnout: A cross-sectional multi-centre study in Switzerland. Health Soc Care Community. 2020;28(6):1989-2003. doi:<https://doi.org/10.1111/hsc.13010>

3. Sexton JB, Helmreich RL, Neilands TB, Rowan K, Vella K, Boyden J, et al. The Safety Attitudes Questionnaire: Psychometric properties, benchmarking data, and emerging research. BMC Health Serv Res. 2006;6:44. doi:10.1186/1472-6963-6-44

4. Zimmermann N, Küng K, Sereika SM, Engberg S, Sexton B, Schwendimann R. Assessing the safety attitudes questionnaire (SAQ), German language version in Swiss university hospitals - a validation study. BMC Health Serv Res. 2013;13(1):347. doi:10.1186/1472-6963-13-347

5. Hart SG, Staveland LE. Development of NASA-TLX (Task Load Index): Results of empirical and theoretical research. Advances in Psychology. 52: Elsevier; 1988. p. 139-83.

6. Schwendimann R, Zúñiga F, Ausserhofer D, Schubert M, Engberg S, Geest S. Swiss Nursing Homes Human Resources Project (SHURP): protocol of an observational study. J Adv Nurs. 2014;70(4):915-26. doi:10.1111/jan.12253

7. Sermeus W, Aiken LH, Van den Heede K, Rafferty AM, Griffiths P, Moreno-Casbas MT, et al. Nurse forecasting in Europe (RN4CAST): rationale, design and methodology. BMC Nurs. 2011;10(1):6. doi:10.1186/1472-6955-10-6

8. Burr H, Berthelsen H, Moncada S, Nübling M, Dupret E, Demiral Y, et al. The Third Version of the Copenhagen Psychosocial Questionnaire. Saf Health Work. 2019;10(4):482-503. doi:<https://doi.org/10.1016/j.shaw.2019.10.002>

9. Orchard CA, King GA, Khalili H, Bezzina MB. Assessment of Interprofessional Team Collaboration Scale (AITCS): development and testing of the instrument. J Contin Educ Health Prof. 2012;32(1):58-67. doi:10.1002/chp.21123

10. Schroder C, Medves J, Paterson M, Byrnes V, Chapman C, O'Riordan A, et al. Development and pilot testing of the collaborative practice assessment tool. J Interprof Care. 2011;25(3):189-95. doi:10.3109/13561820.2010.532620

11. Thomas CL, Spitzmüller C, Amspoker AB, Modi V, Tran T, Naik AD, et al. A Systematic Literature Review of Instruments to Measure Coordination. J Healthc Manag. 2018;63(3):e1-e18. doi:10.1097/jhm-d-16-00025

12. Zackrison EJ. Organizational Coordination and Communication: The Development and Testing of an Integrative Model: UC Santa Barbara; 2017.

13. Gittell JH. Relational coordination: Guidelines for theory, measurement and analysis. Waltham, MA: Brandeis University; 2011.

14. Stalpers D, Kieft R, van der Linden D, Kaljouw MJ, Schuurmans MJ. Concordance between nurse-reported quality of care and quality of care as publicly reported by nurse-sensitive indicators. BMC Health Serv Res. 2016;16:120. doi:10.1186/s12913-016-1372-z

15. Agency for Healthcare Research and Quality. CAHPS Home Health Care Survey. Rockville, MD: Agency for Healthcare Research and Quality; 2018.

16. McGuiness C, Sibthorpe B. Development and initial validation of a measure of coordination of health care. Int J Qual Health Care. 2003;15(4):309-18. doi:10.1093/intqhc/mzg043

17. Haggerty JL, Roberge D, Freeman GK, Beaulieu C, Bréton M. Validation of a generic measure of continuity of care: when patients encounter several clinicians. Ann Fam Med. 2012;10(5):443-51.

18. Bundesamt für Statistik. Schweizerische Gesundheitsbefragung 2017 - Telefonischer und schriftlicher Fragebogen. Neuchâtel: Bundesamt für Statistik.; 2019.
